# Role of caspase-11 non-canonical inflammasomes in retinal ischemia/reperfusion injury

**DOI:** 10.1186/s10020-024-00938-0

**Published:** 2024-09-27

**Authors:** Yong Wan, Jiayu Li, Jialei Pu, Jing Yang, Cheng Pei, Yun Qi

**Affiliations:** 1 Department of Ophthalmology, The First Affiliated Hospital of Xi’an Jiaotong University, Xi’an, 710061 Shaanxi China; 2 Department of Geriatric Surgery, The First Affiliated Hospital of Xi’an Jiaotong University, Xi’an, 710061 Shaanxi China; 3 Department of Health Medicine, The First Affiliated Hospital of Xi’an Jiaotong University, Xi’an, 710061 Shaanxi China

**Keywords:** Caspase-11 non-canonical inflammasomes, Retinal ischemia/reperfusion injury, Interleukin-1β, Caspase-1, GSDMD

## Abstract

**Background:**

Retinal ischemia/reperfusion (IR) injury is a common pathological process in many ophthalmic diseases. Interleukin-1β (IL-1β) is an important inflammatory factor involved in the pathology of retinal IR injury, but the mechanism by which IL-1β is regulated in such injury remains unclear. Caspase-11 non-canonical inflammasomes can regulate the synthesis and secretion of IL-1β, but its role in retinal IR injury has not been elucidated. This study aimed to evaluate the role of caspase-11 non-canonical inflammasomes in retinal IR injury.

**Methods:**

Retinal IR injury was induced in C57BL/6J mice by increasing the intraocular pressure to 110 mmHg for 60 min. The post-injury changes in retinal morphology and function and in IL-1β expression were compared between caspase-11 gene knockout (caspase-11^−/−^) mice and wild-type (WT) mice. Morphological and functional changes were evaluated using hematoxylin–eosin staining and retinal whole mount staining and using electroretinography (ERG), respectively. IL-1β expression in the retina was measured using enzyme-linked immunosorbent assay (ELISA). The levels of caspase-11-related protein were measured using western blot analysis. The location of caspase-11 in the retina was determined via immunofluorescence staining. Mouse type I astrocytes C8-D1A cells were used to validate the effects of caspase-11 simulation via hypoxia in vitro. Small-interfering RNA targeting caspase-11 was constructed. Cell viability was evaluated using the MTT assay. IL-1β expression in supernatant and cell lysate was measured using ELISA. The levels of caspase-11-related protein were measured using western blot analysis.

**Results:**

Retinal ganglion cell death and retinal edema were more ameliorated, and the ERG b-wave amplitude was better after retinal IR injury in caspase-11^−/−^ mice than in WT mice. Further, caspase-11^−/−^ mice showed lower protein expressions of IL-1β, cleaved caspase-1, and gasdermin D (GSDMD) in the retina after retinal IR injury. Caspase-11 protein was expressed in retinal glial cells, and caspase-11 knockdown played a protective role against hypoxia in C8-D1A cells. The expression levels of IL-1β, cleaved caspase-1, and GSDMD were inhibited after hypoxia in the si-caspase-11 constructed cells.

**Conclusions:**

Retinal IR injury activates caspase-11 non-canonical inflammasomes in glial cells of the retina. This results in increased protein levels of GSDMD and IL-1β and leads to damage in the inner layer of the retina.

**Supplementary Information:**

The online version contains supplementary material available at 10.1186/s10020-024-00938-0.

## Background

Retinal ischemia/reperfusion (IR) injury is a common pathological process in many ophthalmic diseases, including retinal artery/vein occlusion, acute glaucoma, retinopathy of premature infants, and ischemic optic neuropathy (Osborne et al. 2004). Retinal IR injury can cause damage to the inner layer of the retina, which can impair visual function (Schmid et al. 2014; Gong et al. 2023). Therefore, the mechanisms of retinal IR injury damage need to be understood to rescue the inner layer of the retina cells and treat the related eye diseases. Inflammation plays a crucial role in retinal IR injury (Abcouwer et al. 2021), with interleukin-1β (IL-1β) being an important inflammatory factor involved in this process (Hangai et al. 1995). After retinal IR injury, IL-1β levels in the retina rapidly increase, leading to cell death in the inner retinal layer (Yoneda et al. 2001; Tun et al. 2019). However, the mechanism by which IL-1β is regulated in retinal IR injury remains unclear. In recent years, the caspase-11 non-canonical inflammasomes has become a research topic of interest in the field of inflammation. Caspase-11 non-canonical inflammasomes promote the maturation and release of IL-1β (Kayagaki et al. 2011), but it is still unclear whether they are involved in retinal IR injury.

Caspase-11, the driving factor for noncanonical inflammasomes, is a protein found in mice, and its homolog caspase-4 is found in humans (Khanova et al. 2018). Caspase-11 non-canonical inflammasomes can regulate the synthesis and secretion of inflammatory cytokines and induce cell death. They activate downstream caspase-1, promoting the release of the mature proinflammatory cytokine IL-1β (Agnew et al. 2021). Moreover, caspase-11 non-canonical inflammasomes activate downstream gasdermin D (GSDMD) proteins; consequently, GSDMD membrane pores are formed, causing pyroptosis (Wu et al. 2021). Many previous studies have shown that caspase-11 non-canonical inflammasomes are activated upon bacterial infection of macrophages (Yi 2017). Thus, the major role of caspase-11 is to protect the host from gram-negative bacterial infections. However, few recent studies have shown that caspase-11 non-canonical inflammasomes can also be activated during sterile damage (Yang et al. 2014; Fagenson et al. 2020; Yin 2022). Retinal IR injury is a sterile injury (Minhas et al. 2016), but it is unclear whether caspase-11 non-canonical inflammasomes are activated during the injury.

Thus, this study aimed to investigate the role of caspase-11 non-canonical inflammasome in retinal IR injury. The results revealed that damage to retinal structure and function caused by retinal IR injury was significantly lesser in caspase-11 gene knockout (caspase-11^−/−^) mice than in WT mice. In vivo and in vitro experiments confirmed the role of caspase-11 non-canonical inflammasomes in retinal IR injury and the mechanisms through which they played their roles. To our best knowledge, this study is the first to report these findings.

## Methods

### Mice

All experiments were approved by the Xi’an Jiaotong University Committee on Animal Care and were carried out following the guidelines set by the Association for Research in Vision and Ophthalmology for the use of animals in ophthalmic and vision research.

Male C57BL/6 J mice weighing between 22 and 25 g were obtained from the Laboratory Animal Centre at Xi’an Jiaotong University. Caspase-11^−/−^ mice were generated by Cyagen Biosciences (Suzhou, China) (Li et al. 2024). The animals were sedated with general anesthesia using an intraperitoneal (i.p.) injection of 10% chloral hydrate (500 mg/kg) before all the procedures. Topical anesthesia was achieved using one drop of 0.5% proparacaine hydrochloride (Santen, Japan). Carbomer (Bausch & Lomb, USA) eye drops were used to prevent corneal dryness. The body temperature was maintained at 37 °C using a homoisothermic heating plate.

### Retinal IR injury experiment

Mice were anesthetized and placed on a homoisothermic heated plate. The anterior chamber of the right eye was cannulated using a 31-gauge needle connected to a normal saline reservoir. The reservoir was elevated to a height of 1.5 m to maintain an intraocular pressure of 110 mmHg, thus causing ischemia, which was confirmed by whitening of the iris and fundus. After 1 h, the height of the infusion bottle was slowly lowered, the needle was removed, and blood flow reperfusion in the iris and fundus was restored. Carbomer was applied to prevent the cornea from drying during and after ischemia. The same method was used for the sham surgery group, but the valve of the infusion device was not opened.

### Quantification of retinal ganglion cells using whole mount staining of RBPMS

The mouse eyeballs were removed and then fixed in 4% paraformaldehyde for 1 h. Subsequently, the cornea, lens, and vitreous body were discarded. The intact retina was peeled off and fixed in 4% paraformaldehyde for another hour and then washed thrice with phosphate-buffered saline (PBS) for 10 min each time. The retina was stored overnight at 4 °C in a PBS solution containing 0.5% Triton, 1% dimethylsulfoxide, and 1% bovine serum albumin. Thereafter, it was washed three times with PBS for 10 min each time and then incubated overnight with rabbit anti-RBPMS antibody (1:200, 15187-1-AP, Proteintech, China). Then, it was washed three times again with PBS for 10 min each time and incubated with the second antibody Alexa Fluor 594 (1:100, AB0151, Abways, China) for 2 h.

After washing with PBS, the retina was incubated with 4ʹ,6-diamidino-2-phenylindole (DAPI, 1:500, BD5010, Bioworld, China) for 20 min and then washed with PBS and plated on a glass slide. The edge of the retina was cut into four lobes to flatten it (another small cut was made in the center of each lobe edge); the lobes were then sealed with 50% glycerol. Fluorescence microscopy images of the retina were obtained. Retinal ganglion cells (RGCs) are not uniformly distributed on the retina, with the number of RGCs varying widely among different regions of the optic disc (Gallego-Ortega et al. 2022; Dräger and Olsen 1981). Therefore, we divided each lobe of the retina into three regions: close range, medium range, and long range. Thus, each retina has 4 lobes, and each lobe has 3 regions. The average value from the 4 lobes for each region was used for statistical analysis.

### Electroretinography

Electroretinography (ERG) was conducted 14 days after retinal IR injury. ERG was performed on the right eyes of the experimental animals according to the standardized protocol of small animal ERG records established by the International Society for Clinical Electrophysiology of Vision. Before ERG, the mice were allowed to adapt to dark environment overnight. Then, they were anesthetized using conventional i.p. injections of 3 mL/kg 1% pentobarbital sodium and 50 µL 10% Lumianning II (Jilin Shengda Pharmaceutical Co., Ltd., China). Tropicamide eye drops (Santen Pharmaceutical Co., Ltd., Japan) were used to dilate the pupils, while 0.5% proparacaine hydrochloride eye drops were used to provide corneal anesthesia. The electrodes were connected properly, with a silver-silver chloride corneal ring electrode used as the action electrode. Stainless steel needle electrodes were used for both the reference and ground electrodes, which were inserted under the subcutaneous tissue of the cheek and tail, respectively. ERG was performed under dim red light at a light intensity of 3.0 cds/m^2^.

### Hematoxylin–eosin staining

Hematoxylin–eosin (HE) staining was conducted 24 h after retinal IR injury. After removing the mouse eyeballs and discarding the cornea, lens, and vitreous body, the optic cup was fixed in 4% paraformaldehyde, dehydrated with gradient alcohol, made transparent with xylene, and soaked in wax before being embedded. The slices were then cut into 4-µm-thick sections and baked. Paraffin sections were dewaxed with xylene and subjected to gradient alcohol hydration. The sections were then washed and stained with hematoxylin (517-28-2, Solarbio, China) for 5 min, followed by rinsing with tap water. Thereafter, the sections were differentiated with hydrochloric acid alcohol for a few seconds. Then, the sections were returned to blue with tap water and stained with eosin (15086-94-9, Shandong Xiya Science Co., Ltd, China) for 5 min. After dehydration with anhydrous alcohol, the sections were exposed to xylene for a few minutes and then sealed with 50% glycerol.

### Immunofluorescence staining

After the mice were anesthetized, their eyeballs were removed and immediately placed in an optimal cutting temperature compound embedding solution (4583, SAKURA, USA). The eyeballs were frozen in liquid nitrogen for 1 min. The freezing slicer was set to cut a slice thickness of 9 μm. The temperature of the cold chamber was maintained at − 25 °C, while that of the slicer was maintained at − 20 °C. During slicing, the optic nerve was cut to a plane parallel to the optic axis, and 6 slices were taken per eyeball. The slices were fixed with 95% ethanol for 5 min and then soaked twice in PBS for 5 min each time. The slices were sealed with 10% AlbuminBovineV (4240GR025, BioFroxx, Germany) and allowed to sit at room temperature for 1 h.

Afterward, the slices were washed thrice with PBS for 3 min each time and then incubated in PBS with the primary antibodies rat anti-Caspase-11 antibody (1:200, ab10454, Abcam) and rabbit anti-GFAP antibody (1:1000, ab7260, Abcam) at room temperature for 1 h. They were then stored in a 4 °C refrigerator overnight. For the secondary antibody incubation, the slices were washed three times with PBS for 3 min each time, and the secondary antibody was then added dropwise. The slices were subsequently incubated at room temperature for 30 min in the dark. Thereafter, they were washed three times with PBS for 3 min each time and finally stained with DAPI (1:1000) for 10 min. Next, they were washed thrice with PBS for 5 min each time and observed and photographed under a fluorescence microscope.

### Western blot analysis

We used distinct protein extraction methods for the retinas and C8-D1A cells. To extract proteins from the detached retinas, the retinas were homogenized for 30 min on ice in a radio-immunoprecipitation assay (RIPA) buffer containing protease inhibitors and phosphatase inhibitors. For C8-D1A cells, the cells were washed three times with PBS and then pipetted repeatedly in the same RIPA lysis buffer. The lysates (retinas or C8-D1A cells) were then centrifuged at 15,000 rpm for 15 min at 4 °C. The supernatant was separated using sodium dodecyl-sulfate polyacrylamide gel electrophoresis (SDS-PAGE), and the pellet was discarded. The protein concentration was determined using the BCA Protein Assay Kit (BIOS, Beijing, China). The protein was mixed with SDS buffer, boiled for 5 min, and then centrifuged at 15,000 rpm for 5 min at 4 °C. The proteins (30 μg) were separated using SDS-PAGE on 12% acrylamide gels and transferred onto a nitrocellulose membrane.

The membranes were blocked with 5% nonfat milk dissolved in Tris-buffered saline supplemented with 0.1% Tween 20 (TBST, pH 7.6) for 1 h and then incubated overnight at 4 °C. The following antibodies were used for incubation: rabbit anti-NLRP3 (1:1000, ab91525, Abcam), rabbit anti-ASC (1:1000, ab70627, Abcam), rabbit anti-GSDMD (1:2000, 20770-1-AP, Proteintech, China), rat anti-caspase-11 (1:500, ab10454, Abcam), rabbit anti-caspase-1 (1:500, ab108362, Abcam), rabbit anti-IL-1β (1:250, ab2105, Abcam), and rabbit anti-β-actin (1:1000, 4970, Cell Signaling Technology, Danvers, MA). After three washes with TBST, the membrane was incubated with a secondary antibody for 1 h at room temperature. The labeled proteins were detected using an enhanced chemiluminescence western blotting system (Pierce Biotechnology, Rockford, USA). The optical density of each band was quantified using Image J analysis software. β-actin was used as the loading control.

### Enzyme-linked immunosorbent assay

The eyes were enucleated at 4, 8, 16, 24, and 48 h after retinal IR injury, and the retinas were carefully isolated, placed in 100 μL PBS, and ground on ice. The lysate was centrifuged at 12,000 rpm for 20 min at 4 °C, and the IL-1β levels in the supernatant were determined using a mouse IL-1β enzyme-linked immunosorbent assay (ELISA) kit (ab197742, Abcam) at 450 nm, with an absorption spectrophotometer (SpectraMax; Bio-Rad, Hercules, CA). The levels were then normalized to the total protein levels according to the manufacturer’s protocol. A standard curve was plotted from measurements of diluted standard solutions, and the IL-1β level in each sample was determined in comparison with this curve.

### Cell culture and the hypoxia model

#### Cell culture

Mouse type I astrocytes C8-D1A (CTCC-001-0937), a murine astrocyte cell line, were acquired from Meisen CTCC (Zhejiang, China). The cells were maintained in high-glucose Dulbecco’s modified Eagle medium (DMEM) supplemented with 10% fetal bovine serum (FBS) and 1% penicillin/streptomycin, all of which were obtained from Gibco (Grand Island, NY, USA). The cells were incubated in a humidified environment with 5% CO_2_ at 37 °C.

#### Establishment of the in vitro C8-D1A cell hypoxia model

C8-D1A cells were digested into a cell suspension, and the cell density was adjusted to 5 × 10^4^ cells/mL. After reaching 80% confluence, the cells were washed 3 times with PBS, the medium was replaced with serum-free DMEM, and the cells were placed in a three-gas incubator containing 1% O_2_/5% CO_2_/94% N_2_ gas mixture at 37 °C to initiate hypoxia for the indicated time.

#### Evaluation of C8-D1A cell viability

Cell viability was assessed using the MTT method. Cell viability was measured using an enzyme-labeling instrument at 492 nm. The hypoxia duration was 3 h and 6 h.

#### Measurement of IL-1β concentration

IL-1β concentrations in samples of culture supernatants and C8-D1A cell lysates were measured using commercial ELISA kits (ab197742, Abcam) according to the manufacturer’s instructions.

#### SiRNA transfection

Small-interfering RNA (SiRNA) targeting caspase-11 was designed and synthesized by Genechem (Shanghai, China). SiRNA-mediated knockdown of caspase-11 expression in C8-D1A cells was performed. The targeted sequence was GTACACGAAAGGCTCTTAT. Recombinant lentivirus was generated from 293T cells. One day before transfection, the cells were seeded at a density of 5 × 10^4^ cells/well onto a 6-well plate. When the cells were growing at 30% confluence, the siRNAs were transfected into the C8-D1A cells following the manufacturer’s protocol. A total of 4 × 10^3^ C8-D1A cells were prepared in a 96-well plate. On the following day, the cells in each well were transduced with packaged recombinant lentivirus at a multiplicity of infection of 100 in DMEM medium containing 10% FBS with 10 μL Polybrene (5 µg/mL). After 12 h, the transduction media were replaced with fresh DMEM with 10% FBS, and incubation was continued for 4 days at 37 °C and 5% CO_2_. The transduced cells were passaged once every three days at a ratio of 1:10. Enhanced green fluorescent protein (GFP) fluorescence, as a marker for evaluating transgene expression of the transduced cells, was examined under a fluorescent microscope. The changes in the gene expression of caspase-11 expression in the control and lentiviral transfection groups were detected using real-time polymerase chain reaction (PCR).

#### Quantitative real-time polymerase chain reaction

Total RNA was collected from C8-D1A cells using a TRIzol kit (Invitrogen, USA). Reverse transcription was performed using the cDNA first-strand synthesis system. The amplification of cDNA samples was determined using the SYBR Green PCR Master Mix (TAKARA, China). The target gene expression was normalized to glyceraldehyde-3-phosphate dehydrogenase. Gene expression was calculated according to the 2 − ∆∆Cq method. The primers were designed and synthesized by Genechem Co., Ltd. (Shanghai, China). The primers for caspase-11 were as follows: forward primer: GGCTACGATGTGGTGGTGAA, reverse primer: GAATGTGCTGTCTGATGTCTGG. The primers for GAPDH were as follows: forward primer: TGGTGAAGGTCGGTGTGAAC, reverse primer: GCTCCTGGAAGATGGTGATGG.

### Statistical analysis

Data are expressed as the mean ± standard error of mean (SEM). Comparisons were made using the one-way analysis of variance test. All statistical analyses were performed using GraphPad software 8.0.1. A p value of < 0.05 was considered significant.

## Results

### Caspase-11 knockout ameliorated RGC death after retinal IR injury

**Fig. 1:**
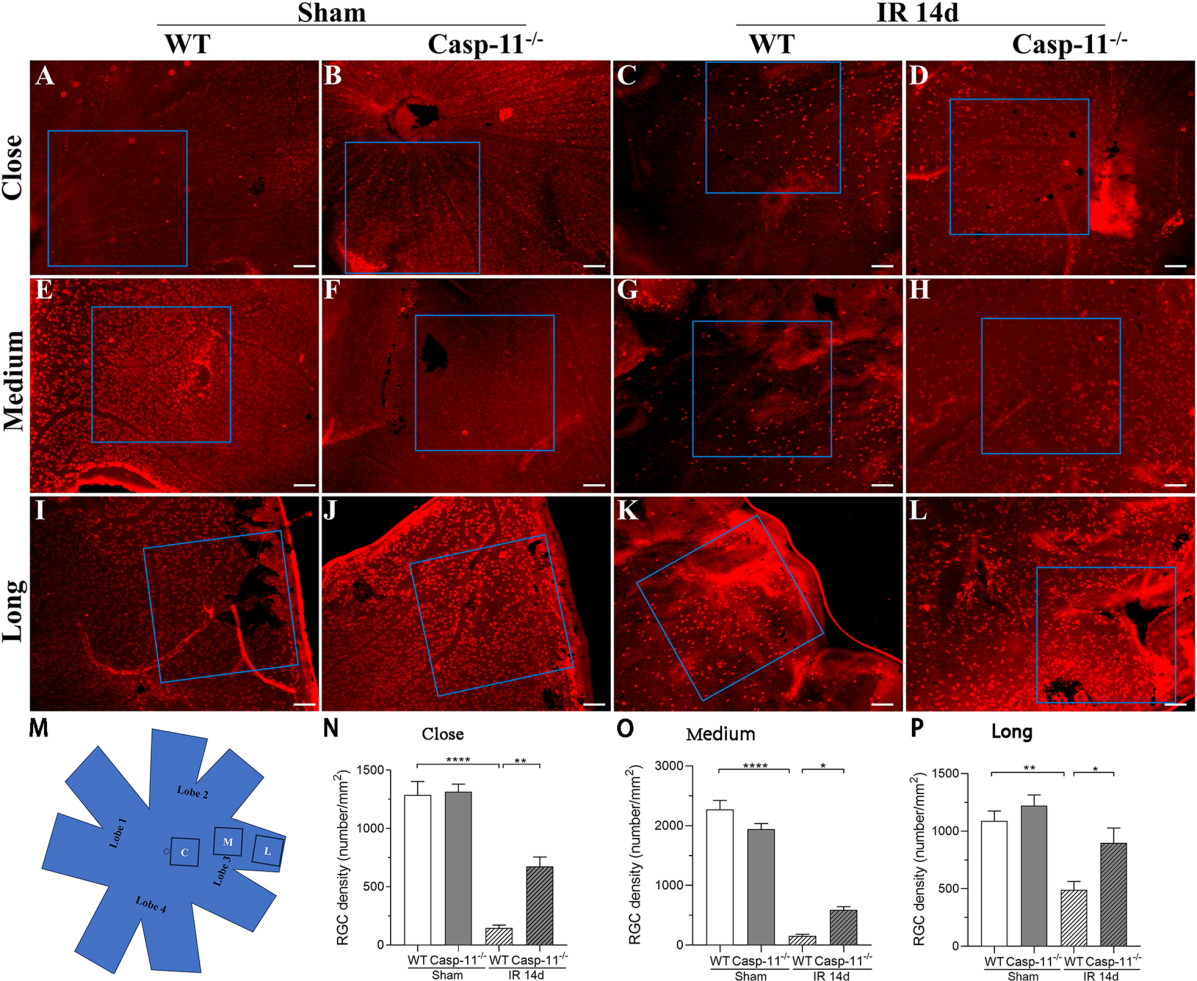
IR-induced RGC death is attenuated in caspase-11^−/−^ mice. Retinas are harvested from mice 14 days after retinal IR or sham injury. **A**–**L** Representative fluorescence micrographs of retinal flat mounts with RBPMS taken in the close, medium, and long-range regions around the optic nerve head, respectively. **M** Schematic diagram of retinal region selection. The retina is cut into four lobes, and cut open again at the edge of each lobe. Three regions (square, 6 mm*6 mm) are established: close to the optic disc (**C**), close to the edge of the retina (**L**), and approximately 1.2 mm from the optic nerve head (**M**). **N**–**P** Density (cells/mm^2^) of RBPMS-labeled RGCs. Data are shown as the mean ± SEM. n = 4 per group. Scale bar = 100 mm. *p < 0.05, **p < 0.01, ****p < 0.0001. *IR* ischemia/reperfusion, *WT* wild type, *Casp-11*^*−/−*^ caspase-11 knockout

The number of RGCs was counted by staining with RBPMS 14 days after IR or sham injury. In the close range region (Fig. 1N), the mean density of RGCs in the WT-sham group was 1288 ± 114 cells/mm^2^ and that in the caspase-11^−/−^-sham group was 1315 ± 66 cells/mm^2^ (p > 0.05 vs. WT-sham). The mean density of RGCs was lower in the WT-IR group at 146 ± 23 cells/mm^2^ (p < 0. 0001 vs. WT-sham), whereas it was higher in the caspase-11^−/−^-IR group at 672 ± 83 cells/mm^2^ (p < 0. 01 vs. WT-IR). These data showed that in the close range region of the retina, the RGC density at 14 days after retinal IR injury was significantly higher in caspase-11^−/−^ mice than in WT mice.

In the medium range region (Fig. 1O), the mean density of RGCs in the WT-sham group was 2273 ± 151 cells/mm^2^ and that in the caspase-11^−/−^-sham group was 1942 ± 94 cells/mm^2^ (p > 0.05 vs. WT-sham). The mean density of RGCs was lower in the WT-IR group at 154 ± 29 cells/mm^2^ (p < 0. 0001 vs. WT-sham), whereas it was higher in the caspase-11^−/−^-IR group at 592 ± 51 cells/mm^2^ (p < 0. 05 vs. WT-IR). These data suggested that in the medium range region of the retina, the RGC density at 14 days after retinal IR injury was significantly greater in caspase-11^−/−^ mice than in WT mice.

In the long range region (Fig. 1P), the mean density of RGCs in the WT-sham mice was 1089 ± 85 cells/mm^2^ and that in the caspase-11^−/−^-sham mice was 1222 ± 92 cells/mm^2^ (p > 0.05 vs. WT-sham). The mean density of RGCs was lower in the WT-IR group at 490 ± 73 cells/mm^2^ (p < 0. 01 vs. WT-sham), whereas it was higher in the caspase-11^−/−^-IR group at 899 ± 127 cells/mm^2^ (p < 0. 05 vs. WT-IR). These data suggested that in the long range region of the retina, the RGC density at 14 days after retinal IR injury was significantly higher in caspase-11^−/−^ mice than in WT mice.

### Caspase-11 knockout improved the ERG b-wave amplitude after retinal IR injury

**Fig. 2:**
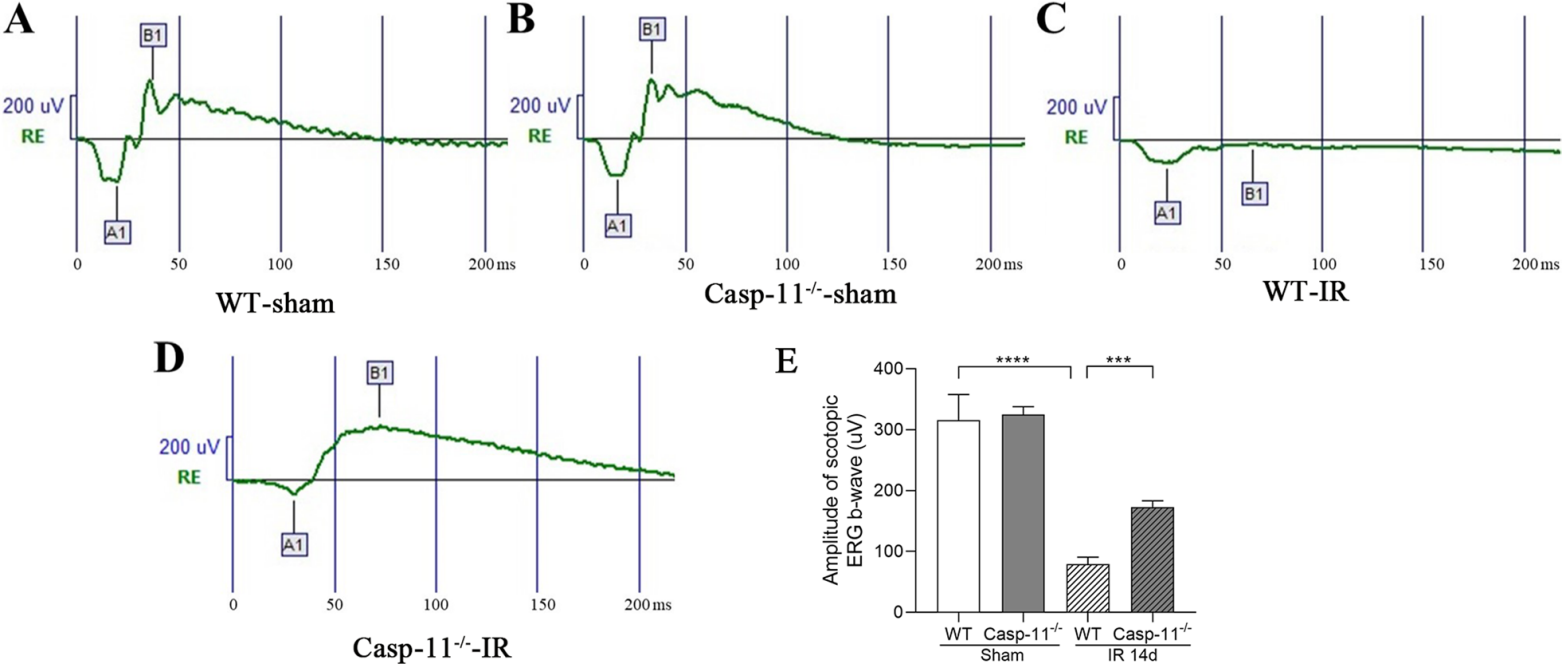
ERG b-wave of different mice groups. **A**–**D** Representative ERG b-wave in different groups. **E** ERG b-wave amplification. The ERG b-wave amplitude is calculated as amplitude A1 + B1. n = 4 per group. ***p < 0.001, ****p < 0.0001. *ERG* electroretinography, *IR* ischemia/reperfusion, *WT* wild type, *Casp-11*^*−/−*^ caspase-11 knockout

The ERG test was conducted to evaluate the electrical activity of retina cells in mice and to assess retinal function (Fig. 2). The results showed that in the WT-Sham and caspase-11^−/−^-sham groups, the amplitudes of the scotopic ERG b-waves were 315 ± 21 µV and 325 ± 6 µV, respectively (p > 0.05). The amplitude of the scotopic ERG b-wave was lower in the WT-IR group at 79 ± 6 µV (p < 0. 0001 vs. WT-sham), whereas it was higher in the caspase-11^−/−^-IR group at 172 ± 5 µV (p < 0. 001 vs. WT-IR). These results indicated that caspase-11 knockout significantly improved the ERG b-wave amplitude 14 days after retinal IR injury.

### Caspase-11 knockout ameliorated retinal edema after retinal IR injury

**Fig. 3:**
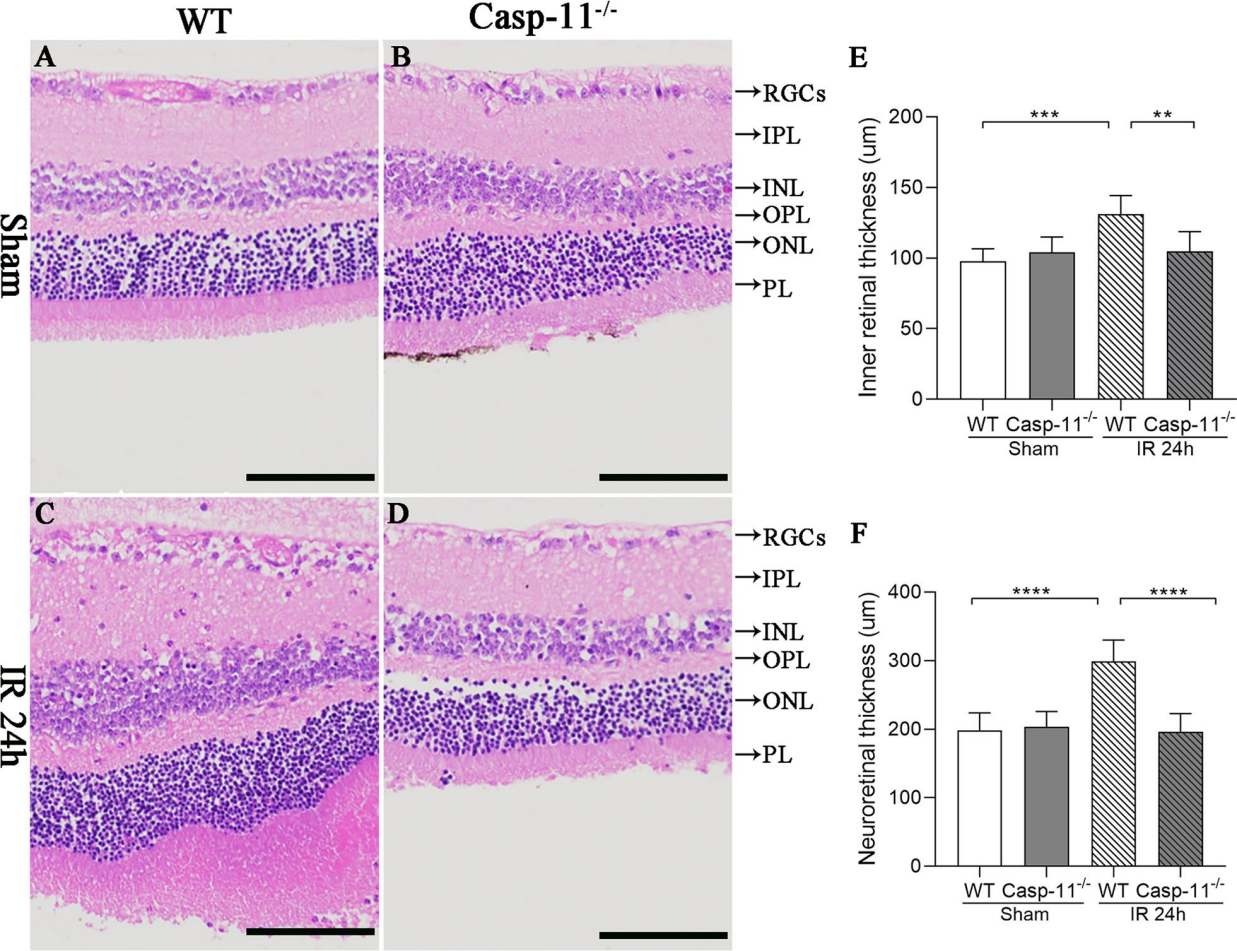
Retinal histology and morphology on HE staining. **A**–**D** Retinal morphology of the different groups. **E** The thickness of the inner retina in the different groups. **F** The thickness of the neuroretina in different groups (n = 6 per group). Scale bar = 100 µm. **p < 0.01. ***p < 0.001, ****p < 0.0001. *IR* ischemia/reperfusion, *WT* wild type, *Casp-11*^*−/−*^ Caspase-11 knockout, *RGCs* retinal ganglion cells, *IPL* inner plexiform layer, *INL* inner nuclear layer, *OPL* outer plexiform layer, *ONL* outer nuclear layer, *PL* photoreceptor layer

In the WT-sham and caspase-11^−/−^-sham groups, the retinas exhibited well-arranged structures on optical microscopy, as shown in Fig. 3. The retina in the WT-IR group exhibited edema, especially in the RGC layer, inner plexiform layer (IPL), and photoreceptor layer (PL). However, there was no significant edema in the retinas in the caspase-11^−/−^-IR group. The thickness of the inner retina, defined as the distance between the inner membrane and the outer plexiform layer (OPL), was also not significantly different between the WT-sham and caspase-11^−/−^-sham groups (97.8 ± 3.6 µm vs 104.1 ± 4.4 µm). The inner retinal thickness was significantly higher in the WT-IR group than in the WT-Sham group (131.3 ± 5.3 µm, p < 0.001). In contrast, the inner retinal thickness was significantly lower in the caspase-11^−/−^-IR group than in the WT-IR group (104.8 ± 5.7 µm, p < 0.01).

With respect to the thickness of the complete neuroretina (defined as the distance between the inner membrane and the PL), no significant difference was found between the WT-Sham and caspase-11^−/−^-sham groups (198.6 ± 10.3 µm vs 203.5 ± 9.1 µm). However, the neuroretina thickness was significantly greater in the WT-IR group than in the WT-sham group (298.9 ± 12.8 µm, p < 0.0001). In contrast, the neuroretina thickness was significantly lower in the caspase-11^−/−^-IR group than in the WT-IR group (196.6 ± 10.7 µm, p < 0.0001).

### Caspase-11 knockout decreased retinal IL-1β expression after retinal IR injury

**Fig. 4:**
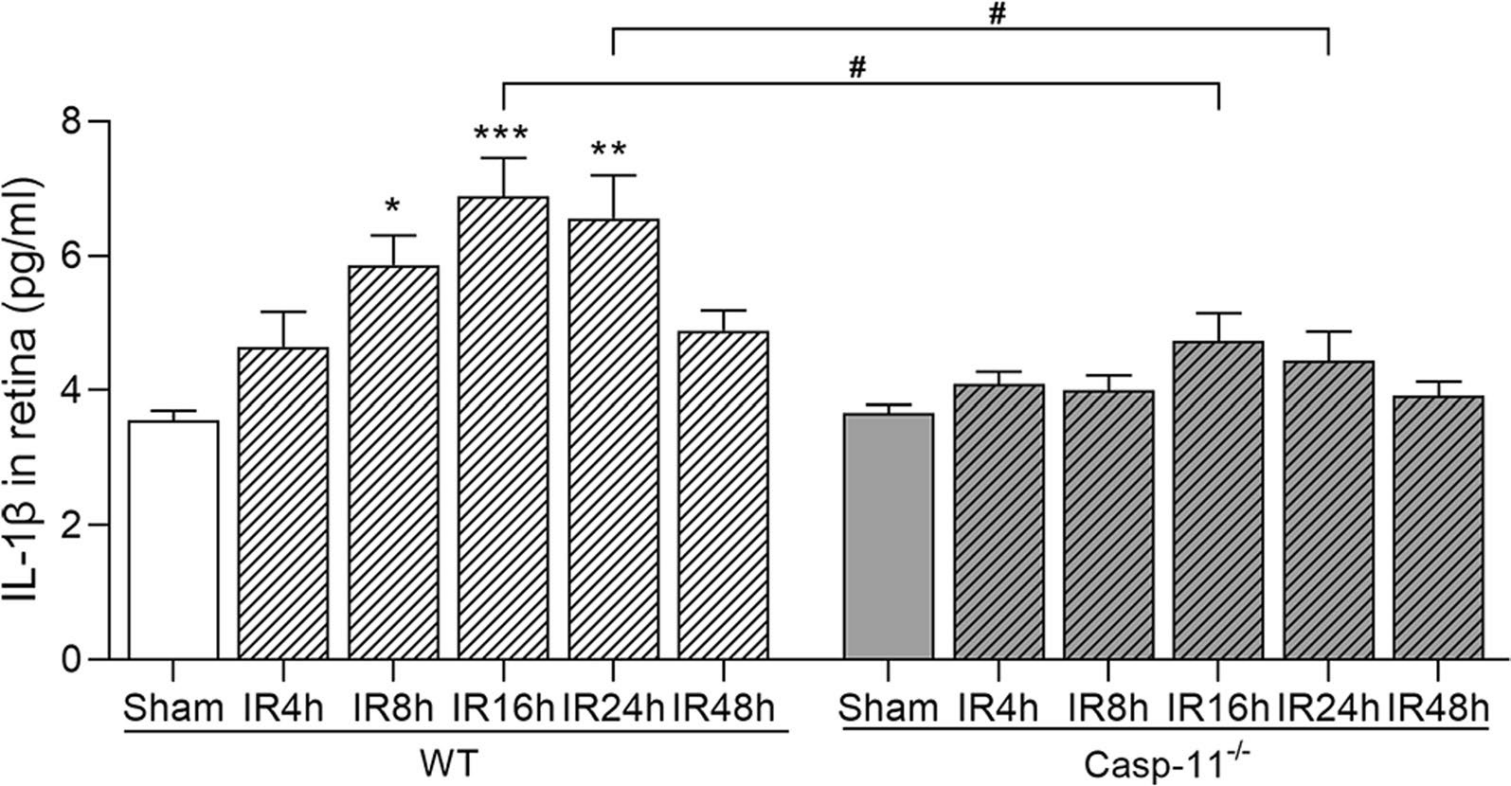
ELISA of IL-1β expression in the WT and caspase-11^−/−^ groups. Values at 4, 8, 16, 24, and 48 h after IR injury in the WT and caspase-11^−/−^ groups are shown. Data are shown as the mean ± SEM. n = 5 per group. *p < 0.05, **p < 0.01, ***p < 0.001, #p < 0.05

As shown in Fig. 4, ELISA results indicated that the protein expressions of IL-1β in the retina at 8, 16, and 24 h after retinal IR injury were significantly higher in the WT group than in the sham group (p < 0.05, p < 0.001, and p < 0.01, respectively). IL-1β expression peaked at 16 h after IR injury. Retinal IL-1β expression was not significantly changed at 4, 8, 16, 24, and 48 h after retinal IR injury in the caspase-11^−/−^ group. Compared with the WT group, the caspase-11^−/−^ group showed significantly lower retinal IL-1β expression at both 16 and 24 h after retinal IR injury (both p < 0.05).

### Caspase-11 protein expression was upregulated after retinal IR injury

**Fig. 5:**
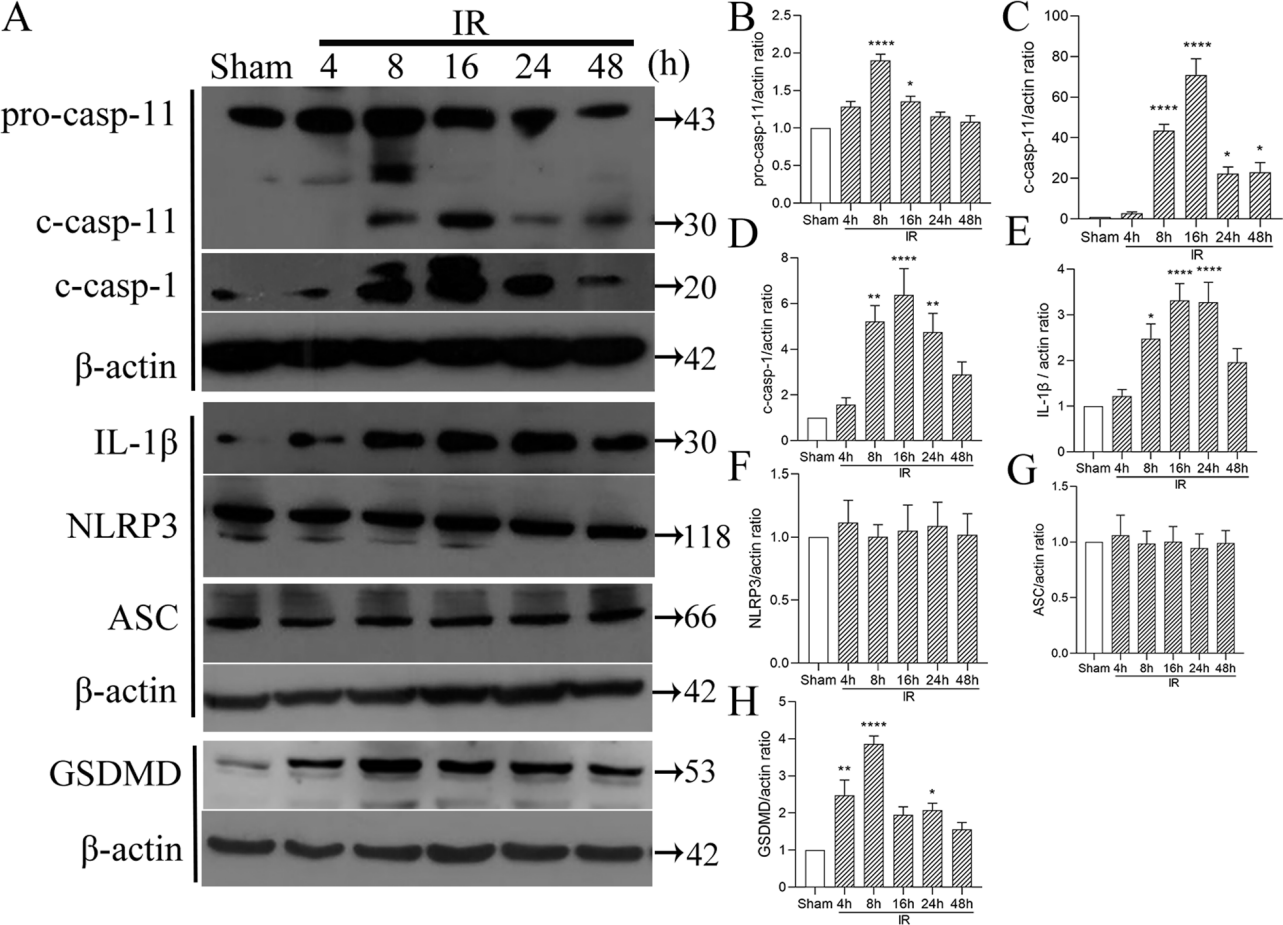
Western blot analysis of caspase-11 protein expression. **A** Representative image of the western blot analysis of caspase-11 protein expression 4, 8, 16, 24, and 48 h after retinal IR injury. **B**–**H** The results of the statistical analysis. Data are shown as the mean ± SEM. n = 5 per group. *p < 0.05, **p < 0.01. ***p < 0.001, ****p < 0.0001. *IR* ischemia/reperfusion, *c-casp-11* cleaved caspase-11, *c-casp-1* cleaved caspase-1

Western blot analysis of the expression of caspase-11 and related proteins showed that the protein expression of retinal pro-caspase-11 was significantly increased at 8 h and 16 h after retinal IR injury (Fig. 5). Cleaved caspase-11 protein expression was significantly increased at 8, 16, 24, and 48 h after retinal IR injury. Pro-caspase-11 and cleaved caspase-11 protein expressions peaked at 8 h and 16 h after retinal IR injury, respectively. With respect to the expression of caspase-11-related proteins, cleaved caspase-1, IL-1β, and GSDMD proteins were highly expressed after retinal IR injury. However, there was no significant change in the protein expression of NLRP3 or ASC after retinal IR injury.

Immunohistochemistry studies to determine the location of caspase-11 in the retina showed that caspase-11 was widely distributed in various layers of the retina, including the ganglion cell layer (GCL), IPL, INL, OPL, and PL (Fig. S1). Caspase-11 protein expression was higher at 8 h after IR injury than in the sham group, consistent with the western blot results.

### Protein expression in caspase-11^−/−^ mice after retinal IR injury

**Fig. 6:**
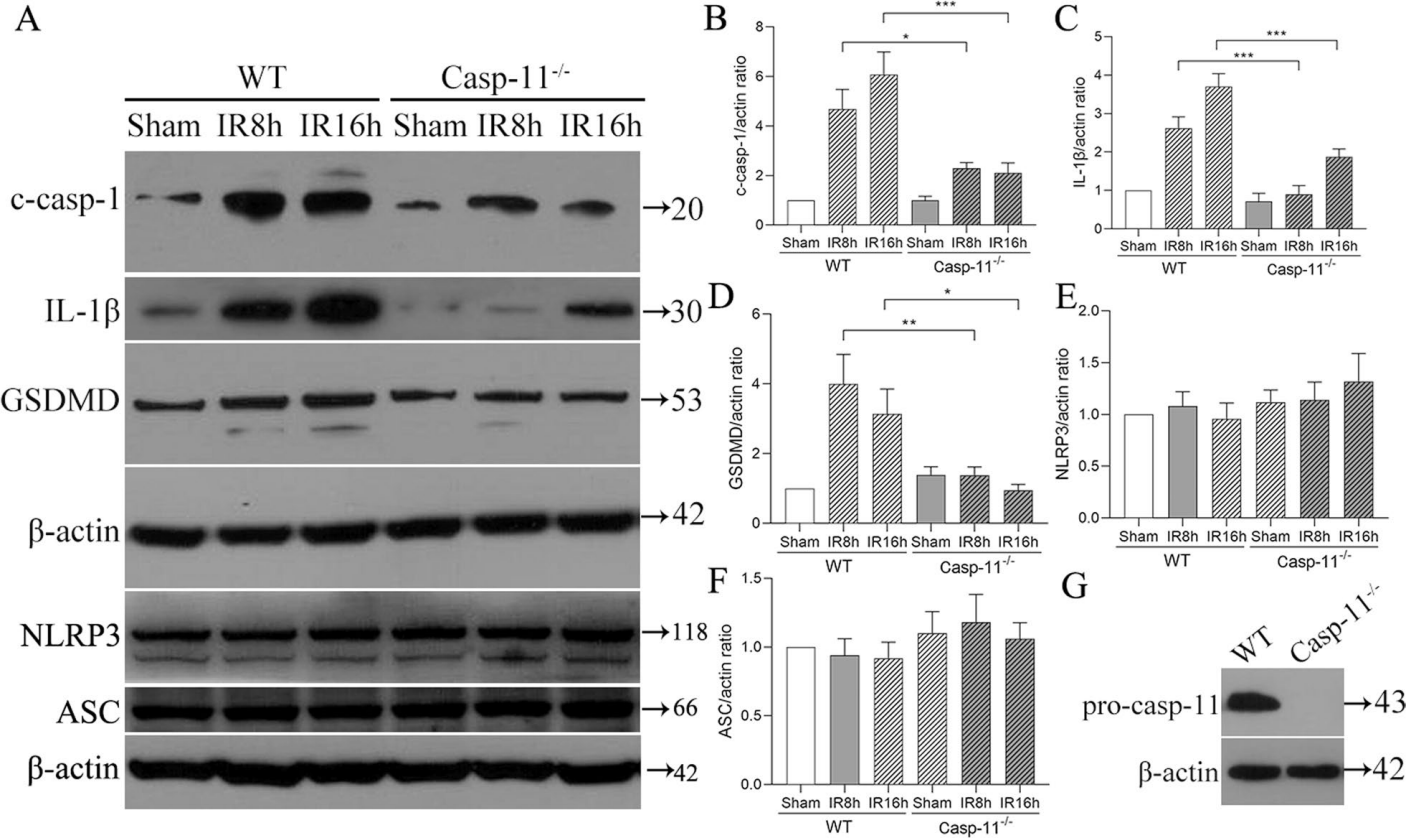
Protein expression in the WT and caspase-11^−/−^ groups. **A** Representative western blot analysis image of caspase-11 protein expression 8 h and 16 h after retinal IR injury. **B**–**F** Results of the statistical analysis. **G** Identification of mice genotype by Western blotting assay. Data are shown as the mean ± SEM. n = 5 per group. *p < 0.05, **p < 0.01. ***p < 0.001. *IR* ischemia/reperfusion, *WT* wild type, *Casp-11*^*−/−*^ Caspase-11 knockout, *c-casp-1* cleaved caspase-1

Comparison of the change in the expression of proteins related to caspase-11 non-canonical inflammasome between the caspase-11^−/−^ and WT groups showed that the expression of caspase-11 related proteins (cleaved caspase-1, IL-1β) peaked at 8 h and 16 h after retinal IR injury (Fig. 5). Thus, further analyses were conducted in these two time points. As shown in Fig. 6, the protein expressions of cleaved caspase-1 and GSDMD at 8 h and 16 h after retinal injury were significantly lower in caspase-11^−/−^ mice than in WT mice. Further, IL-1β expression at 8 h after retinal IR injury was also significantly lower in caspase-11^−/−^ mice. The protein expression of NLRP3 or ASC was not significantly changed at 8 h and 16 h after retinal IR injury in either caspase-11^−/−^ mice or WT mice.

### Caspase-11 protein was expressed in retinal glial cells

Glial cells are widely present in the retina and play a crucial role in its structure (Bringmann and Wiedemann 2011; Newman and Zahs 1998; Goldman 2014). When the retina is damaged because of injury, glial cells can become activated and produce inflammatory factors that can damage nerve cells (Bianchi et al. 2015; Bringmann et al. 2005). To investigate whether caspase-11 was expressed in retinal glial cells, we performed immune co-staining of GFAP and caspase-11 proteins in the IR-damaged retina. GFAP and caspase-11 were co-stained in the sham group. Given that caspase-11 expression peaked at 8 h after retinal IR injury, we chose 8 h after retinal IR injury as the time point for immune co-staining. As shown in Figure S2, the co-staining sites of caspase-11 and GFAP remained the same as those in the sham group, and caspase-11 expression was increased at 8 h after retinal IR injury.

### Caspase-11 knockdown protected against hypoxia in C8-D1A cells

To investigate the role of caspase-11 non-canonical inflammasomes following hypoxia in retinal glial cells, we cultured C8-D1A cells (an astrocyte glial cell line) in vitro and transfected them with si-caspase-11. As shown in Figure S3, caspase-11 was knockdown in C8-D1A cells transfected with SiRNA. MTT colorimetry revealed that compared to that of the control group, cell viability was significantly decreased 3 h and 6 h after hypoxia (both p < 0.05, Fig. S4A). Compared with that in the hypoxia group, cell viability was significantly greater at both 3 h and 6 h after hypoxia in the si-caspase-11 transfected group (p < 0.05 and p < 0.01, respectively). Western blot analysis revealed that compared with that in the hypoxia group, the expression levels of pro-caspase-11, cleaved caspase-1, IL-1β, and GSDMD were significantly lower at both 3 h and 6 h after hypoxia in the si-caspase-11 transfected group (Fig. S4D).

We also measured IL-1β expression in both cells lysate and supernatant using ELISA. As shown in Figures S4B and S4C, compared with that in the hypoxia group, IL-1β expression was inhibited in both the cell lysate and supernatant of C8-D1A cells 3 h and 6 h after hypoxia in the si-caspase-11 transfected group. Overall, our findings suggest that caspase-11 knockdown plays a protective role against hypoxia in C8-D1A cells, decreasing the activation of c-caspase-1, IL-1β and GSDMD. This result confirms the role of caspase-11 non-canonical inflammasomes in glial cells. Hypoxia injury increased the expression of caspase-11 in glial cells. The caspase-11 non-canonical inflammasome then promoted the expression of GSDMD, caspase-1, and IL-1β, thus damaging glial cells. Moreover, hypoxia injury increased IL-1β expression in the supernatant of glial cells, which may cause inflammatory damage to surrounding cells such as RGCs.

## Discussion

The major novel findings of the present study can be summarized as follows. Retinal IR injury activates caspase-11 non-canonical inflammasomes in glial cells of the retina, increasing the protein expressions of GSDMD, cleaved caspase-1, and IL-1β and resulting in damage to the inner retinal layer. To our best knowledge, this study is the first to confirm the role of caspase-11 non-canonical inflammasomes in retinal IR injury and the mechanisms through which they were involved.

Retinal IR injury can cause severe damage to the structure and function of the retina, and inflammation plays an important role in this injury process. Retinal IR injury can cause a transient increase in IL-1β expression after retinal IR injury (Yoneda et al. 2001; Tun et al. 2019). Treatment with an anti-IL-1β antibody before the onset of ischemia reduces damage (Yoneda et al. 2001). Our results indicate that retinal IR injury leads to the upregulation of IL-1β expression during the early stage of retinal IR injury (8–24 h), consistent with the findings reported by Liu et al. (Liu et al. 2021). IL-1β production is controlled by caspase-11 non-canonical or NLRP3 canonical inflammasome pathways (Kayagaki et al. 2011; Schmidt and Lenz 2012). To determine whether they regulated IL-1β in the retina after IR injury, we used caspase-11 gene knockout mice. The results revealed that after caspase-11 knockout, IL-1β expression after retinal IR injury was significantly lower in caspase-11^−/−^ mice than in WT mice. However, the protein expressions of NLRP3 and ASC did not significantly change after retinal IR injury. This result indicates that in the early stages of retinal IR injury, IL-1β is mainly produced by the caspase-11 non-canonical inflammasome pathway rather than by the NLRP3 canonical inflammasome pathway in the retina.

Caspase-11 non-canonical inflammasomes activate the downstream GSDMD protein, causing pyroptosis (Wu et al. 2021). In our study, we compared caspase-11^−/−^ mice with WT mice. After 14 days of retinal IR injury, the retinal whole mount results showed a significantly higher number of RGCs in caspase-11^−/−^ mice than in WT mice. The ERG results showed that the damage to retinal visual function was more significantly alleviated in caspase-11^−/−^ mice than in WT mice. These results indicate that caspase-11 non-canonical inflammasomes play an important role in retinal IR injury. This finding is interesting. In a study by Pronin In A et al. (Pronin et al. 2019), there was no difference in the number of RGCs between caspase-11 gene knockout mice and WT mice at 24 h after retinal IR injury. We studied the changes in RGCs and visual function 14 days after IR, whereas they studied the changes in RGCs on day 1 after IR. On the first day after retinal IR injury, RGC death occurred in the primary stage. The number of RGCs death stabilize after 2–3 weeks of reperfusion (Lafuente et al. 2002). Thus, it is more appropriate to evaluate RGC injury after 2 weeks of reperfusion. Second, we used three methods for evaluation, namely, retinal whole mount staining, which could be used to comprehensively evaluate the number of RGCs; ERG, which could be used to evaluate the degree of visual functional damage; and HE, which could be used to evaluate the degree of retinal edema. However, their study only used HE staining for evaluating RGC injury. HE staining only produces local fault sections and cannot be used to comprehensively evaluate the overall number of RGCs. Thus, our evaluation method is more comprehensive. In addition, in a study by Galina et al. (Dvoriantchikova et al. 2023), GSDMD gene expression in the retina increased compared to that in the control group at 6 h and 24 h after IR injury. Consistent findings were found in the current study (Fig. 5). GSDMD is a downstream molecule of caspase-11 non-canonical inflammasomes (Shi et al. 2015). This finding also confirms, to a certain extent, the activation of caspase-11 non-canonical inflammasomes after IR injury.

We confirmed the activation of caspase-11 non-canonical inflammasomes in retinas with IR injury using western blot and immunofluorescence staining. Caspase-11 non-canonical inflammasomes are activated when bacteria infect macrophages (Yi 2017). Activation of caspase-11 non-canonical inflammasomes is triggered by lipopolysaccharide from gram-negative bacteria. Activated caspase-11 non-canonical inflammasomes directly cleave GSDMD. Moreover, caspase-11 non-canonical inflammasomes activate downstream caspase-1, promoting the release of the mature proinflammatory cytokine IL-1β (Agnew et al. 2021). Recent studies have shown that sterile injuries, including in renal and liver IR injuries, can also activate caspase-11 non-canonical inflammasomes (Yang et al. 2014; Fagenson et al. 2020; Yin 2022). Our immunofluorescence staining results (Fig. S1) showed that caspase-11 non-canonical inflammasomes were widely distributed in various layers of the retina, including in the GCL, IPL, INL, OPL, and PL. The protein expression of caspase-11 in the retina peaked at 8 h after retinal IR injury. The expressions of GSDMD and IL-1β were also significantly increased. After caspase-11 knockout, the protein expressions of GSDMD and IL-1β at 8 h and 16 h after retinal IR injury were significantly lower in caspase-11^−/−^ mice than in WT mice (Fig. 6). This result indicates that GSDMD and IL-1β are downstream proteins of caspase-11 non-canonical inflammasomes in retinal IR injury. To the best of our knowledge, this is the first study confirming the role and the mechanism of caspase-11 non-canonical inflammasome in retinal IR injury. It is currently unclear how retinal IR, a sterile injury, activates caspase-11 non-canonical inflammasomes. Further research is needed.

We used co-immunostaining for GFAP, a highly specific marker for glial cells, and caspase-11 and found that caspase-11 was highly expressed in glial cells in the retina (Fig. S2). The number of caspase-11 non-canonical inflammasomes were significantly increased in glial cells after retinal IR injury. Previous studies reported that caspase-11 non-canonical inflammasomes are mainly activated in immune cells, including macrophages (Yi 2017) and neutrophils (Kovacs et al. 2020). Retinal glial cells are a source of innate immune cells (Kumar et al. 2013; Pan et al. 2021), and this can explain the role of caspase-11 non-canonical inflammasomes in the retina.

In addition, studies have shown that the astrocyte glial cell caspase-11 non-canonical inflammasomes can be activated by IR injury in the brain (Fradejas et al. 2010). Therefore, we used a glial cell line (C8-D1A cells) for further validation. Hypoxia promoted the protein expressions of caspase-11, GSDMD, IL-1β, and IL-1β in the supernatant of C8-D1A cells. After caspase-11 gene silencing, the protein expressions of caspase-11, GSDMD, and IL-1β in C8-D1A cells, as well as that of IL-1β in the cell supernatant, were significantly downregulated after hypoxia. IL-1β secreted by retinal glial cells can cause inflammatory death in RGCs (Fernández-Albarral et al. 2021; Wooff et al. 2019). Therefore, we speculate that IL-1β secreted in the supernatant of C8-D1A cells after hypoxia may act on surrounding RGCs, causing inflammatory damage to RGCs.

**Fig. 7:**
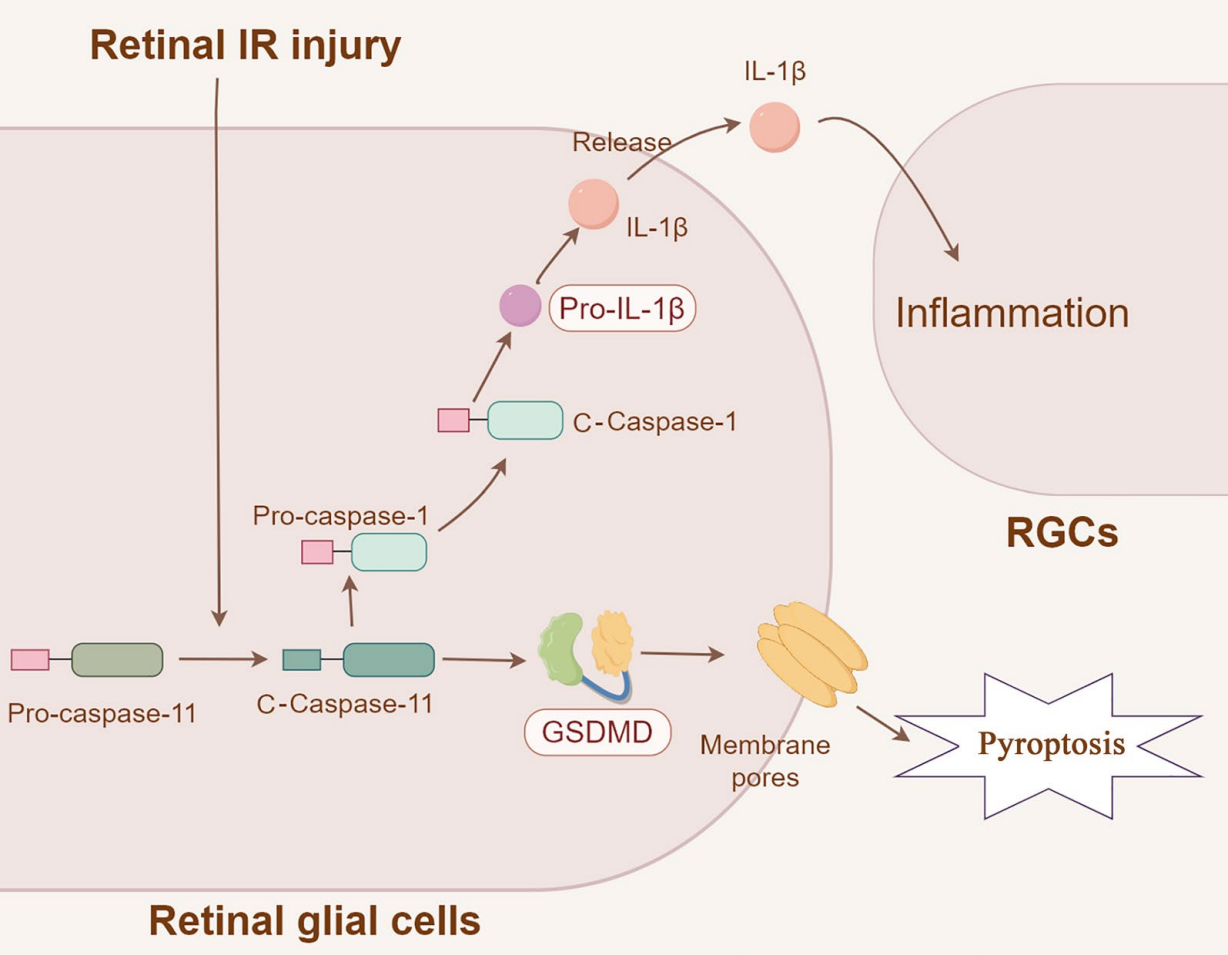
Schematic showing the mechanism of caspase-11 activation. Retinal IR injury activates caspase-11 in retinal glial cells, and caspase-11 activates downstream caspase-1, IL-1β, and GSDMD. Formation of GSDMD membrane pores causes pyroptosis. IL-1β secreted into the extracellular space causes inflammatory injury in RGCs. *IR* ischemia/reperfusion, *RGCs* retinal ganglion cells, *c-caspase-1* cleaved caspase-1, *c-caspase-11* cleaved caspase-11

The mechanism by which caspase-11 non-canonical inflammasomes are involved in retinal IR injury is shown in Fig. 7. Retinal IR injury activates caspase-11 non-canonical inflammasomes in retinal glial cells, and caspase-11 non-canonical inflammasomes in turn activate downstream caspase-1, IL-1β, and GSDMD. GSDMD causes pyroptosis. IL-1β secreted into the extracellular space may cause inflammatory injury in RGCs. Information regarding inflammation in retinal IR injury has markedly increased over the past decade. However, the molecular mechanisms underlying inflammation in retinal IR injury have not been fully elucidated. The present study not only reveals for the first time that caspase-11 non-canonical inflammasomes are involved in the retinal IR injury, but also identifies the downstream related molecules. Inhibition of caspase-11 expression may become a therapeutic strategy for treating IR-related ophthalmic diseases. Besides, only few studies reported that sterile damage can activate caspase-11 non-canonical inflammasomes. Our study showed that retinal IR, a sterile injury, activated caspase-11 non-canonical inflammasomes, thus confirming a new function of caspase-11.

Finally, we studied the mechanism by which caspase-11 non-canonical inflammasomes played a role in the early stage of IR, but whether they were also involved in the later stage of IR is unclear. In vitro experiments in which C8-D1A cells are cocultured with RGCs are needed to further clarify the mechanism of RGC death. Moreover, further human experiments are needed to verify the role of caspase-11.

## Conclusions

Our study confirms the role and mechanism of caspase-11 non-canonical inflammasomes in retinal IR injury. Retinal IR injury activates caspase-11 non-canonical inflammasomes in glial cells of the retina, causing an increase in GSDMD and IL-1β protein expressions and resulting in damage to the inner retinal layer.

## Supplementary Information

Additional file 1: Fig. S1. Immunofluorescence staining of caspase-11/DAPI in retinal cross sections. A. Caspase-11/DAPI staining in the sham group. B. Caspase-11/DAPI staining 8 hours after retinal IR injury. Green fluorescence represents caspase-11 staining. Blue fluorescence represents the nucleus. Scale bar = 50 μm. IR, ischemia/reperfusion; Casp-11, Caspase-11; RGCs, retinal ganglion cells; IPL, inner plexiform layer; INL, inner nuclear layer; OPL, outer plexiform layer; ONL, outer nuclear layer; PL, photoreceptor layer.

Additional file 2: Fig. S2. Co-immunofluorescence staining for DAPI/GFAP/Caspase-11 in retinal cross sections. (A) DAPI/GFAP/caspase-11 co-staining in the sham group. (B) DAPI/GFAP/caspase-11 co-staining 8 hours after retinal IR injury. Blue fluorescence represents the nucleus. Red fluorescence represents GFAP staining. Green fluorescence represents caspase-11 staining. Yellow fluorescence represents GFAP and caspase-11 co-staining. Scale bar = 50 μm. IR, ischemia/reperfusion; RGCs, retinal ganglion cells; IPL, inner plexiform layer; INL, inner nuclear layer; OPL, outer plexiform layer; ONL, outer nuclear layer; PL, photoreceptor layer.

Additional file 3: Fig. S3. Lentiviral-mediated transduction of GFP green fluorescence into C8-D1A cells. (A) High GFP expression (>90%) was shown in the transfection cells. (B) Identification of Caspase-11 mRNA by RT-PCR assay in C8-D1A cells with or without si-caspase-11 transfection. CON, control. SiCasp11, siRNA-mediated knockdown of Casp-11 expression in C8-D1A cells.

Additional file 4: Fig. S4. Effect of different treatments on C8-D1A cells. (A) Measurement of C8-D1A cell viability (MTT assay). (B-C) ELISA analyses of IL-1β expression in the supernatant and cell lysate of C8-D1A cells after hypoxia. (D) Western blot analysis of protein expression in C8-D1A cells after hypoxia. (E-H) Results of the statistical analysis of the western blot. *p < 0.05, **p < 0.01. SiCasp-11, siRNA-mediated knockdown of Casp-11 expression in C8-D1A cells; c-casp-1, cleaved caspase-1.

## Data Availability

The data that support the findings of this study are available from the corresponding author upon reasonable request.

## Abbreviations

IR: Ischemia/reperfusion
IL-1β: Interleukin-1β
caspase-11^−^^/^^−^: Caspase-11 gene knockout
WT: Wild-type
RGC: Retinal ganglion cell
ERG: Electroretinogram
siRNA: Small-interfering RNA
ARVO: Association for Research in Vision and Ophthalmology
i.p.: Intraperitoneal
HE: Hematoxylin–eosin
TTBS: Tris-buffered saline supplemented with Tween
ANOVA: One-way analysis of variance
IPL: Inner plexiform layer
PL: Photoreceptor layer
OPL: Outer plexiform layer
GCL: Ganglion cell layer
