# Supplementary figures and images for: Role of caspase-11 non-canonical inflammasomes in retinal ischemia/reperfusion injury
Source: Mol Med. 2024 Sep 27;30:159. doi: 10.1186/s10020-024-00938-0 (PMC11429960; doi:10.1186/s10020-024-00938-0)

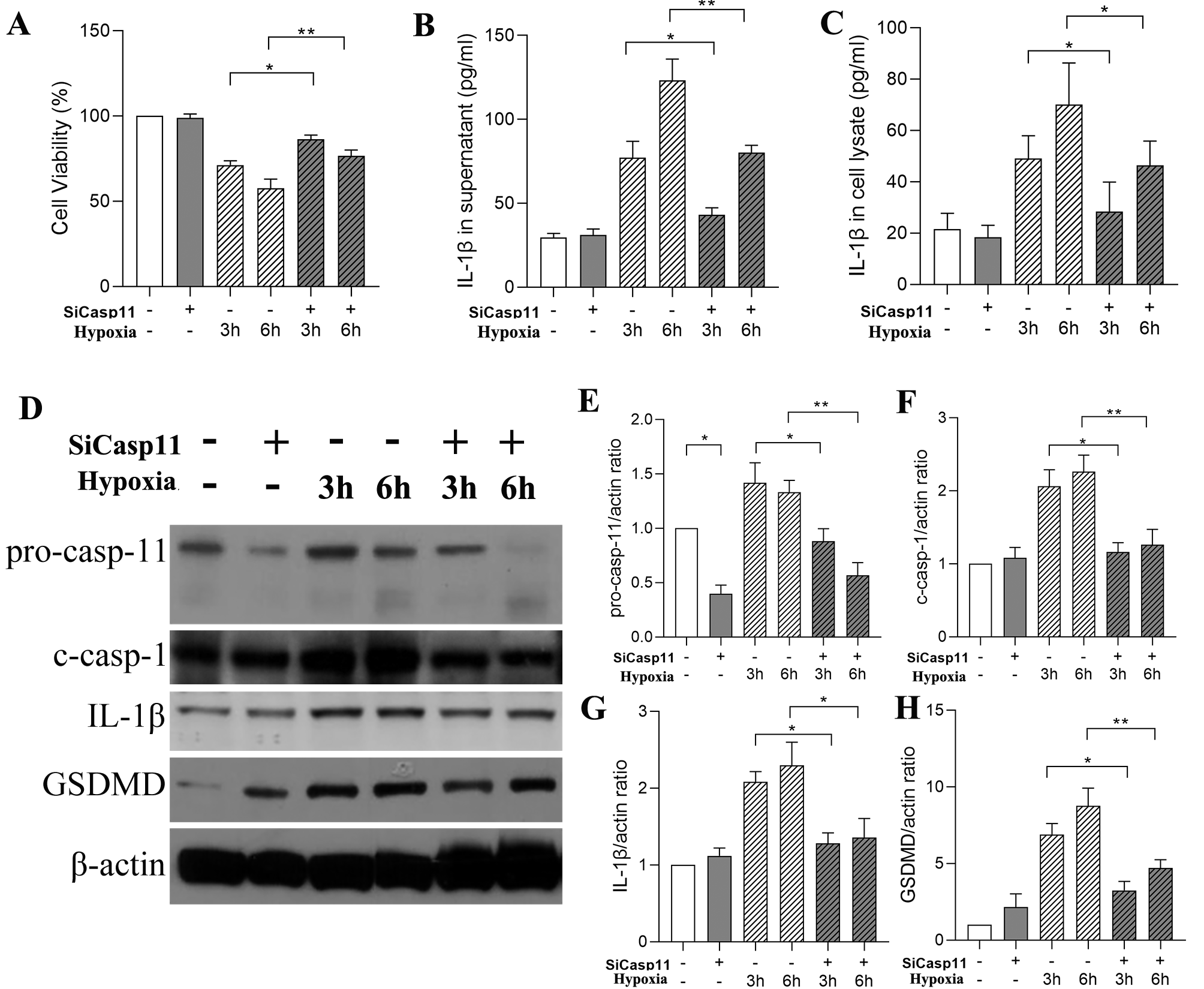

Supplement: Supplementary file 4 — Additional file 4: Fig. S4. Effect of different treatments on C8-D1A cells. (A) Measurement of C8-D1A cell viability (MTT assay). (B-C) ELISA analyses of IL-1β expression in the supernatant and cell lysate of C8-D1A cells after hypoxia. (D) Western blot analysis of protein expression in C8-D1A cells after hypoxia. (E-H) Results of the statistical analysis of the western blot. *p < 0.05, **p < 0.01. SiCasp-11, siRNA-mediated knockdown of Casp-11 expression in C8-D1A cells; c-casp-1, cleaved caspase-1. [file 10020_2024_938_MOESM4_ESM.tif]
